# Sustained Egr-1 Response via p38 MAP Kinase Signaling Modulates Early Immune Responses of Dendritic Cells Parasitized by *Toxoplasma gondii*

**DOI:** 10.3389/fcimb.2019.00349

**Published:** 2019-10-11

**Authors:** Arne L. ten Hoeve, Mohamed-Ali Hakimi, Antonio Barragan

**Affiliations:** ^1^Department of Molecular Biosciences, The Wenner-Gren Institute, Stockholm University, Stockholm, Sweden; ^2^Institute for Advanced Biosciences, INSERM U1209, CNRS UMR5309, Université Grenoble Alpes, Grenoble, France

**Keywords:** transcription factor, CD40, CD80, host-pathogen, intracellular signaling, apicomplexa

## Abstract

As a response to a diverse array of external stimuli, early growth response protein 1 (Egr-1) plays important roles in the transcriptional regulation of inflammation and the cellular immune response. However, a number of intracellular pathogens colonize immune cells and the implication of Egr-1 in the host-pathogen interplay has remained elusive. Here, we have characterized the Egr-1 responses of primary murine and human dendritic cells (DCs) upon challenge with the obligate intracellular parasite *Toxoplasma gondii*. We report that live intracellular parasites induce a sustained high expression of Egr-1 in DCs, different from the immediate-early Egr-1 response to parasite lysates, inactivated parasites or LPS. Moreover, a distinct nuclear localization of elevated amounts of Egr-1 protein was detected in infected DCs, but not in by-stander DCs. The ERK1/2 MAPK signaling pathway mediated the canonical immediate-early Egr-1 response to soluble antigens in a MyD88/TLR-dependent fashion. In contrast, a non-canonical extended Egr-1 response that relied primarily on p38 MAPK signaling was induced by intracellular parasites and was exhibited similarly by MyD88-deficient and wildtype DCs. The extended phase Egr-1 response was dramatically reduced upon challenge of DCs with *T. gondii* parasites deficient in GRA24, a secreted p38-interacting protein. Further, *Egr-1-*silenced primary DCs maintained their migratory responses upon *T. gondii* challenge. Importantly, *Egr-1* silencing led to elevated expression of co-stimulatory molecules (CD40, CD80) in Toxoplasma-infected DCs and in LPS-challenged immature DCs, indicating that Egr-1 responses suppressed maturation of DCs. Moreover, the IL-12 and IL-2 responses of Toxoplasma-challenged DCs were modulated in a GRA24-dependent fashion. Jointly, the data show that the Egr-1 responses of DCs to microbial external stimuli and intracellular stimuli can be selectively mediated by ERK1/2 or p38 MAPK signaling, and that Egr-1 can act as an intrinsic negative modulator of maturation in primary DCs.

## Introduction

Leukocytes populate the blood and traffic the tissues, draining to the lymphatic system and back into the circulation (Friedl and Weigelin, 2008). As antigen presenting cells, DCs capture and process antigens in the periphery in an immature state and upon maturation migrate to lymphoid organs and act as potent activators of T cell-mediated immunity (Banchereau and Steinman, 1998). Paradoxically, these precise migratory features of DCs make them also opportunistic targets for intracellular pathogens to mediate their dissemination in the host (Santiago-Tirado and Doering, 2017; Bhandage et al., 2019).

Following oral ingestion of the common parasite *Toxoplasma gondii*, inflammatory monocytic cells, such as DCs are recruited to the site of invasion in the intestine (Cohen and Denkers, 2015). Thus, infectious *Toxoplasma* tachyzoite stages exploit DCs for dissemination via a “Trojan horse” mechanism (Courret et al., 2006; Lambert et al., 2006). When actively invaded by *T. gondii*, DCs adopt a hypermigratory phenotype (reviewed in Weidner and Barragan, 2014), which mediates rapid systemic dissemination of *Toxoplasma* in mice (Lambert et al., 2006; Kanatani et al., 2017). This dramatic migratory activation requires the discharge of parasitic secretory organelles into the host cell cytoplasm (Weidner et al., 2013) and intracellular signaling (Fuks et al., 2012; Kanatani et al., 2017). It has also recently become clear that *T. gondii* actively targets host gene expression by releasing effectors into the host cell and modulating signaling pathways and transcription factor activity (Hakimi et al., 2017). Along these lines, challenge of DCs with *T. gondii* tachyzoites induces maturation events, e.g., moderate elevation of co-stimulatory molecules and MHC class II, albeit less pronounced than LPS-induced maturation (McKee et al., 2004; Lambert et al., 2006; Fuks et al., 2012), and *T. gondii* infection renders parasitized DCs refractory to maturation signals (McKee et al., 2004). However, differences in responses have been reported for human and murine DCs and between DC subsets (Subauste and Wessendarp, 2000; Tosh et al., 2016) and the molecular mechanisms for how active invasion by the parasite modulates maturation have remained elusive.

The Early growth response (Egr) proteins are a family of four zinc-finger transcription factors (Sukhatme, 1990). Its founding member Egr-1 is rapidly and transiently induced in response to diverse stimuli, such as serum, growth factors and radiation injury, and has been attributed pleiotropic functions, e.g., in cell growth, differentiation, and apoptosis (Gashler and Sukhatme, 1995). Aberrant Egr-1 expression has been linked to multiple human diseases, including cancer, ischemic injury, vascular disease, and inflammation (Bhattacharyya et al., 2013). In B and T cells, Egr-1 functions as a positive regulative factor of activation, while Egr-2 and 3 have been attributed opposite effects (Gomez-Martin et al., 2010). Less is known on the role of Egr-1 in myeloid lineages. Although not essential for monocytic differentiation, Egr-1 promotes differentiation at the expense of the granulocytic lineage (Krishnaraju et al., 2001; Carter and Tourtellotte, 2007). Mitogen-activated protein kinase (MAPK) signaling has been implicated in the regulation of Egr-1 responses (Lim et al., 1998; Guha et al., 2001). While dendritic cell (DC) maturation has been also linked to MAPK signaling (Puig-Kroger et al., 2001), the role of Egr-1 in DC maturation has remained unelucidated.

Here, we have addressed the impact of Egr-1 responses and MAPK signaling in primary human and murine DCs upon challenge with live *T. gondii* and other external stimuli (*T. gondii* lysates and LPS). We found that intracellular *T. gondii* promotes a sustained high expression Egr-1 in DCs, with maintained migratory responses of DCs. Further, we report that Egr-1 responses are tightly and selectively regulated by MAPK signaling in DCs with down-modulatory effects on DC maturation.

## Materials and Methods

### Ethical Statement

The Regional Animal Research Ethical Board, Stockholm, Sweden, approved protocols involving extraction of cells from mice, following proceedings described in EU legislation (Council Directive 2010/63/EU). The Regional Ethics Committee, Stockholm, Sweden, approved protocols involving human cells. All donors received written and oral information upon donation of blood at the Karolinska University Hospital. Written consent was obtained for utilization of white blood cells for research purposes.

### Cell Culture

Murine bone marrow-derived DCs (BMDCs) were generated as described previously (Olafsson et al., 2018). Cells from bone marrow of wild-type (WT) or MyD88^−/−^ 6–10-weeks-old male or female C57BL/6 mice (Charles River) were cultivated in RPMI 1640 with 10% fetal bovine serum (FBS), gentamicin (20 μg/ml), glutamine (2 mM), and HEPES (0.01 M), referred to as complete medium (all reagents from Life Technologies), and supplemented with 20 ng/ml recombinant mouse GM-CSF (Peprotech). Medium was replenished every other day. Loosely adherent cells were harvested after 6–9 days of differentiation, depending on experiment.

Human monocyte-derived DCs (MoDCs) were generated as previously described (Olafsson et al., 2018). Briefly, monocytes were isolated using RosetteSep (StemCell Technologies) and density gradient centrifugation on Lymphoprep (Axis Shield Poc As) from buffy coat obtained from healthy donors at the Karolinska University Hospital Blood Center. The purified cells were cultured 5–7 days in DMEM high glucose (Invitrogen) with 10% fetal bovine serum (FBS), gentamicin (20 μg/ml, Gibco), glutamine (2 mM, Gibco) and HEPES (0.01 M, Gibco), complete medium, supplemented with 100 ng/ml GM-CSF (Peprotech) and 12.5 ng/ml IL-4 (R&D Systems).

Human foreskin fibroblasts (HFF-1 SCRC-1041, American Type Culture Collection) were cultured in Dulbecco's modified Eagle's medium, high glucose (DMEM; Thermo Fisher scientific), with 10% fetal bovine serum (FBS; Sigma), gentamicin (20 μg/ml; Gibco), glutamine (2 mM; Gibco), and HEPES (0.01 M; Gibco), referred to as DMEM.

### Parasite Culture

*Toxoplasma gondii* tachyzoites of the wild-type dsRed-expressing Prugniaud strain (PRU-RFP, type II) (Pepper et al., 2008), GFP- and luciferase-expressing Ptg strain (PTG, type II) (Olafsson et al., 2018), GFP-expressing RH-LDM (type I) (Barragan and Sibley, 2002), and knockout Prugniaud Δku80 (PRUku80, type II) and Prugniaud Δku80 Δgra24 (PRUΔgra24, type II) (Braun et al., 2013) lines were maintained by serial 2 days passages in human foreskin fibroblast monolayers. Given how readily *Egr-1* is induced by a variety of stimuli, we minimized the carry-over from routine *T. gondii* culture to experiments by repeated washing of the tachyzoites before preparation of the heat-inactivated tachyzoites and lysates (sonicated tachyzoites) or challenge with live tachyzoites.

### Inhibitors

Trametinib (Selleckchem) was used at 1 μM and BIRB 796 (Doramapimod, Calbiochem) at 10 μM, dissolved in DMSO. Other conditions were treated with the DMSO vehicle.

### Quantitative Polymerase Chain Reaction (qPCR)

For *Egr-1* and *Egr-2* kinetics qPCR analysis WT BMDCs (2 × 10^6^) were cultured with CM and challenged with freshly egressed *T. gondii* tachyzoites (PTG) for 30 min or 1 h (MOI 5) or 2, 4, 8, or 12 h (MOI 3), the equivalent number of sonicated *T. gondii* tachyzoites or heat-inactivated *T. gondii* tachyzoites or LPS 10 ng/mL or left unchallenged. For *Egr-1* kinetics qPCR analysis WT or MyD88^−/−^ BMDCs (5 × 10^5^) were cultured with CM or challenged with freshly egressed *T. gondii* tachyzoites (PTG) for 1 (MOI 5), 2, 4, or 8 h (MOI 3). For all other qPCR analysis BMDCs (5 × 10^5^) were cultured with CM or indicated inhibitors and challenged with freshly egressed *T. gondii* tachyzoites for 1, 8 h (PRUku80 or PRUku80 Δgra24; MOI 3), with heat-inactivated tachyzoites (MOI 3 equivalents) or LPS 10 ng/mL or 24 h (RH-LDM, PRU-RFP, or PTG; MOI 1). Cells were lysed in TRIzol reagent (Thermo Fisher Scientific) or TRI Reagent (Sigma-Aldrich) and total RNA was extracted using column purification according to the manufacturers protocol (Direct-zol RNA kit, Zymo Research). Reverse transcription was conducted with Superscript III or IV or Maxima H Minus Reverse Transcriptase (Thermo Fisher). Real time qPCR was performed in duplicate for each target using sing SYBR® green PCR master mix (KAPA biosystems), specific forward and reverse primers at target-dependent concentrations (100 or 200 nM) and cDNA (10–25 ng) in a Rotor-Gene 6000 (Corbett) or a QuantStudio 5 System (Thermo Fisher) with ROX as a passive reference. qPCR results were analyzed using the ΔΔCq method and displayed as fold change relative to non-challenged or as ΔCq and relative to Glyceraldehyde 3-phosphate dehydrogenase and β-actin (mouse) or Importin-8 and TATA-binding protein (human) as housekeeping genes. Primers have been described previously or were designed manually or with Primer3 (Olafsson et al., 2018). Sequences are provided in Table S1.

### Western Blotting

For western blotting WT BMDCs (2 × 10^6^) or confluent HFFs were cultured in complete medium and challenged with freshly egressed *T. gondii* tachyzoites, equivalent amount of sonicated or heat-inactivated *T. gondii* tachyzoites, at indicated MOI, or LPS 10 ng/mL. When indicated, treatment with inhibitors preceded challenge by 45 min. BMDCs were harvested and washed with PBS and then lysed directly in Laemmli buffer for whole cell lysates or lysed in cytoplasmic extraction buffer (10 mM HEPES, 60 mM KCl, 0.5 mM DTT, 0.05% Triton X-100 and cOmplete mini protease and phosphatase inhibitors, Roche) after which nuclei were separated as described (Miller et al., 1998) by centrifugation, washed and lysed in Laemmli buffer. Proteins were separated using 8% or 10% SDS-PAGE, blotted onto a PVDF membrane and blocked (10% BSA) followed by incubation with primary and secondary antibodies: anti-TATA-binding protein (TBP; ab51841, abcam), anti-EGR1 (15F7, Cell Signaling), anti-phosho-p38 (D3F9, Cell Signaling), anti-mouse IgG-HRP (32430, Thermo Fisher), or anti-rabbit IgG-HRP (7074S, Cell Signaling). Proteins were revealed by mean of enhanced chemiluminescence (GE Healthcare) in a BioRad ChemiDoc XRS^+^. Densitometry analysis was performed using ImageJ (NIH, MD, USA).

### Immunofluorescence

BMDCs (3 × 10^4^) were seeded on poly-L-lysine (Sigma) -coated glass coverslips and challenged with freshly egressed *T. gondii* tachyzoites (PTG; MOI 1, infection frequencies ranged between 40 and 60%), the equivalent number of sonicated *T. gondii* tachyzoites or heat-inactivated *T. gondii* tachyzoites or LPS 10 ng/mL for 8 h. After fixation with 2% PFA (Sigma) and blocking and permeabilization with 5% FBS/0.1% Triton X-100, cells were stained with anti-Egr-1 (clone 15F7, Cell Signaling) and anti-rabbit Alexa Fluor 594 (A-21442, Invitrogen) antibodies and DAPI. Images were acquired on a Zeiss Observer Z1 with 20x objective or Leica DMi8 with 63x objective with identical settings of each condition and analyzed with ImageJ (Schneider et al., 2012). For quantification of Egr-1-expressing cells, a threshold was set and defined as the mean of the 3x 20 brightest pixels from 3 fields of view with cells stained with DAPI and anti-rabbit Alexa Fluor 594 only. Cellular signals above the threshold were considered positive.

### Gene Silencing by Short Hairpin RNA (shRNA)

Lentivirus for transduction was produced as reported previously (Olafsson et al., 2018), with modifications, as follows. Briefly, LentiX 293T cells (Clontech) or HEK 293T cells were transfected with self-inactivating lentiviral vectors for expression of reporter GFP under a CMV-promoter and short hairpin RNA targeting luciferase or murine Egr-1 (Table S1) under a U6 promoter, pLL3.7-shLuc (described previously Kanatani et al., 2017) and pLKO.1-shEgr1 (TRCN0000231216, Sigma), and psPAX2 (12260, Addgene) packaging plasmid and pCMV-VSVg (8454, Addgene) envelope plasmid with Lipofectamine 2000 (Invitrogen). The resulting supernatant was harvested 24 and 48 h post-transfection. Recovered lentivirus-containing supernatants were centrifuged to eliminate cell debris, filtered through 0.45 μm filters and used immediately or aliquoted and kept at −80°C. Bone marrow cells cultured in the presence of GM-CSF, for 2 days (on average 5.6% ± 0.5 CD11c^+^ as determined with flow cytometry) for flow cytometry experiments or 5 days for motility and qPCR analysis, were transduced with lentivirus-containing supernatant in the presence of DEAE-dextran (10 μg/mL; Sigma) or treated with DEAE-dextran alone for 4 h followed by washing with CM and further culture in the presence of GM-CSF. The expression of GFP was confirmed by epifluorescence microscopy repeatedly after transduction and before cells were used in experiments after a total of 8 days in culture.

### Motility Assays

Motility assays were performed as described previously and briefly as follows. BMDCs were cultured in 96-well plates in CM with and without freshly egressed *T. gondii* tachyzoites (PRU-RFP or CMTMR dye (Invitrogen)-stained WT or Δgra24 PRUku80, MOI 3, 4 h). Bovine collagen type I (1 mg/mL, Life Technologies) was then added and live cell imaging was performed for 1 h, 1 frame/min, at 10 × magnification on a Zeiss Z1 Observer. Time-lapse images were consolidated into stacks and motility data was obtained from at least 40 cells/condition/experiment (Manual Tracking, ImageJ) yielding mean velocities (Chemotaxis and migration tool v2.0, Ibidi). Infected cells were defined by RFP-cell co-localization. Transduced cells were defined by GFP reporter expression.

### Flow Cytometry

For phenotypic analysis cell from BMDC cultures were harvested by repeated pipetting and centrifuged at 350 g to include moderately adherent and smaller cells. The cells were then blocked one ice in FACS buffer (1% FBS and 0.5 mM EDTA in PBS) with anti-CD16/CD32 antibody (Fc Block, BD Pharmingen). Cells were then stained with anti-CD11c (clone N418, Biolegend), CD11b (clone M1/70, Biolegend), MHCII I-A/I-E (clone M5/114, Invitrogen), CD40 (clone 1C10, Invitrogen), CD80 (clone 16-10A1, Invitrogen) and/or CD86 (clone GL1, Invitrogen) antibodies in FACS buffer, fixed with 2% PFA and analyzed on a BD LSRFortessa flow cytometer (BD Biosciences). For maturation experiments BMDCs were challenged with freshly egressed *T. gondii* tachyzoites (PRU-RFP, MOI 1, infection frequencies ranged between 15 and 30%) or 100 ng/mL LPS or left unchallenged for 24 h.

### Statistical Analyses

Statistical analyses were performed with R version 3.4.3 and RStudio version 1.1.383. The area under the curve was calculated with the R package PK with bootstrapping. For statistical comparisons the tests indicated were used as implemented in the R packages stats, PMCMRplus, dunn.test and PK. Multiple comparisons of normally distributed data were carried out with one-way ANOVA followed by Tukey HSD (all-to-all comparisons) or Dunnett's (all-to-one comparisons) *post-hoc* tests. Non-parametric ANOVA (Kruskal-Wallis) followed by a Dunn's *post-hoc* test was used for multiple comparisons of non-parametric data. Two sample comparisons were carried out with the non-parametric Mann-Whitney test. A non-parametric permutation test was used for area under the curve comparisons.

## Results

### Challenge of DCs With *T. gondii* Induces a Biphasic *Egr-1* mRNA Expression and Nuclear Translocation of *Egr-1* Protein in Parasitized DCs

**Figure 1:**
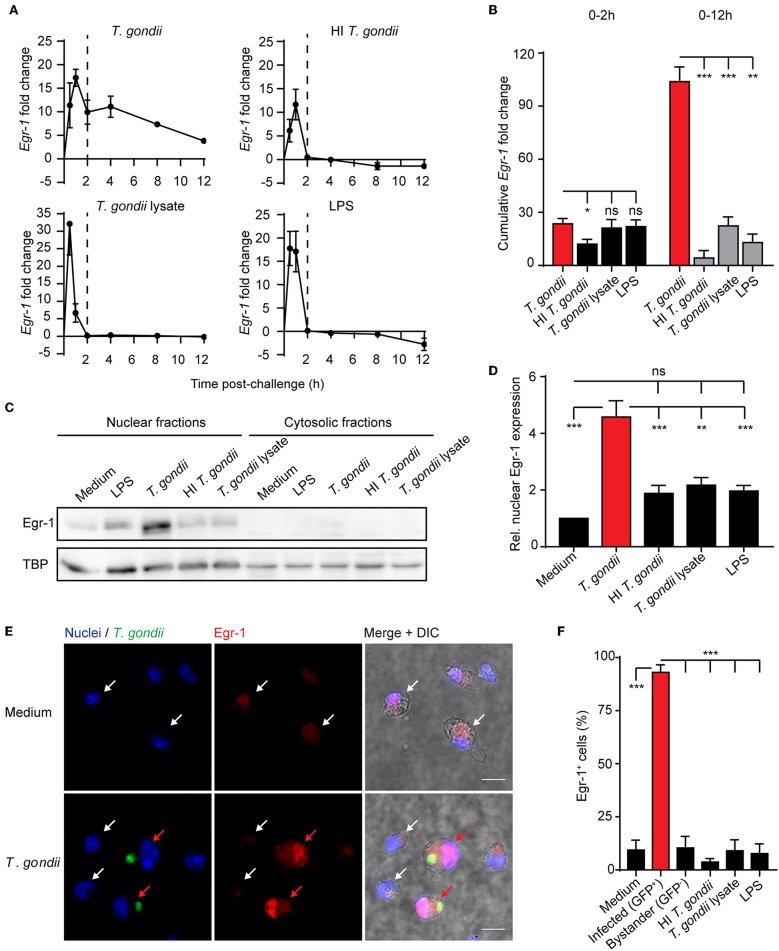
*Egr-1* mRNA expression and nuclear localization of Egr-1 in BMDCs challenged with live *T. gondii* or lysates. **(A)** qPCR analysis of Egr-1 cDNA from BMDCs challenged with freshly egressed *T. gondii* tachyzoites (PTG), LPS 10 ng/mL, heat-inactivated (HI) tachyzoites, or tachyzoite lysate for the indicated time related to unchallenged BMDCs in complete medium (CM). Each timepoint represents the mean ± SEM of 3 independent experiments. The dashed line indicates 2 h timepoint. **(B)** Area under the curve analysis of the graphs displayed under **(A)** for the first 2 h or the whole period. Bars indicate, for each condition, the cumulative *Egr-1* fold change ± SE (*p ≤ 0.05, **p ≤ 0.01, ***p ≤ 0.001, ns *p* > 0.05, permutation test). **(C)** Representative Western blot of nucleus- and cytosol-enriched fractions of BMDCs challenged with freshly egressed *T. gondii* tachyzoites (PTG), LPS 10 ng/mL, heat-inactivated (HI) tachyzoites, tachyzoite lysate for 8 h, or left unchallenged in CM. **(D)** Densitometric analysis of Western blots, as in **(C)**, displayed as the mean ± SEM from 3 independent experiments (**p ≤ 0.01, ***p ≤ 0.001, ns *p* > 0.05, ANOVA, Dunnett's). **(E)** Representative micrographs of BMDCs challenged with freshly egressed GFP-expressing *T. gondii* tachyzoites (PTG, MOI 1) for 8 h, stained for Egr-1 (red) and with DAPI (blue). Red arrows indicate infected DCs (green). White arrows indicate by-stander DCs or unchallenged DCs. Scale bar 10 μm. **(F)** Frequency of BMDCs with detectable Egr-1 expression. Unchallenged BMDCs (medium), BMDCs challenged at MOI 1 and infected with *T. gondii* tachyzoites (PTG, GFP^+^/Alexa 594^+^) related to the total infected population (GFP/ Alexa 594^+/−^), bystander non-infected BMDCs (GFP^−^/Alexa 594^+^) related to the total by-stander population (GFP^−^/Alexa 594^+/−^) or challenged with LPS (10 ng/mL), heat-inactivated tachyzoites or tachyzoite lysate. Relative expression was determined by pixel threshold analysis as indicated under Materials and Methods. Plots show the mean ± SEM from 3 independent experiments (***p ≤ 0.001, ANOVA, Dunnett's).

While the implication of *Egr-1* in the regulation of the inflammatory response is well established (McMahon and Monroe, 1996), its roles in the host cell-pathogen interplay has remained elusive (Braun et al., 2013). To address this, murine bone marrow-derived DCs (BMDCs) were challenged with freshly egressed *T. gondii* tachyzoites. Upon challenge with LPS, lysate from *T. gondii* tachyzoites or heat-inactivated tachyzoites, BMDCs displayed a rapid and relatively short-lived (2 h) *Egr-1* mRNA response (Figure 1A). In contrast, BMDCs challenged with live *T. gondii* tachyzoites presented a biphasic up-regulation of *Egr-1* mRNA expression, with an immediate-early response, and an extended response that persisted for at least 12 h after challenge (Figure 1A). Consequently, highly significant differences in the cumulative fold change of *Egr-1* mRNA expression were measured for *Toxoplasma*-challenged BMDCs compared with heat-inactivated parasites, parasite lysate or LPS, while non-significant or minor differences were measured during the immediate-early phase (Figure 1B). Next, we sought to determine protein expression and subcellular localization of Egr-1 in the extended phase. Western blotting analyses at 8 h post-challenge detected signal corresponding to Egr-1 primarily in nucleus-enriched fractions (Figure 1C). Importantly, the relative signal from nucleus-enriched fractions was significantly stronger in lysates from BMDCs challenged with live *T. gondii* compared with other conditions (Figures 1C,D). This relative Egr-1 protein expression difference was absent in the early phase (Figure S1), in line with transcriptional data (Figure 1B). Importantly, immunofluorescence staining confirmed a strong Egr-1 signal with nuclear localization in Toxoplasma-infected BMDCs, which was absent in by-stander or unchallenged BMDCs (Figures 1E,F). This indicated that the global *Egr-1* mRNA upregulation observed in *T. gondii*-challenged BMDCs was mirrored in nuclear Egr-1 protein levels in *T. gondii*-infected BMDCs but not in by-stander BMDCs. In line with the above, *Egr-1* up-regulation upon *T. gondii* challenge was also induced in human foreskin fibroblasts (HFFs) (Figure S2A), and in BMDCs by different lines of *T. gondii* (type I and II) (Figures S2B,C). A similar, but less pronounced, expression pattern was also observed for *Egr-2* (Figure S2D). Altogether, the data show that early Egr-1 expression is primarily induced by soluble *T. gondii*-derived factors/inactivated parasites/LPS while an extended Egr-1 expression phase was primarily a response of BMDCs to live intracellular *T. gondii*.

### The Extended Phase *Egr-1* Response of DCs to *T. gondii* Is MyD88-, TLR11-, and TLR12-independent

**Figure 2:**
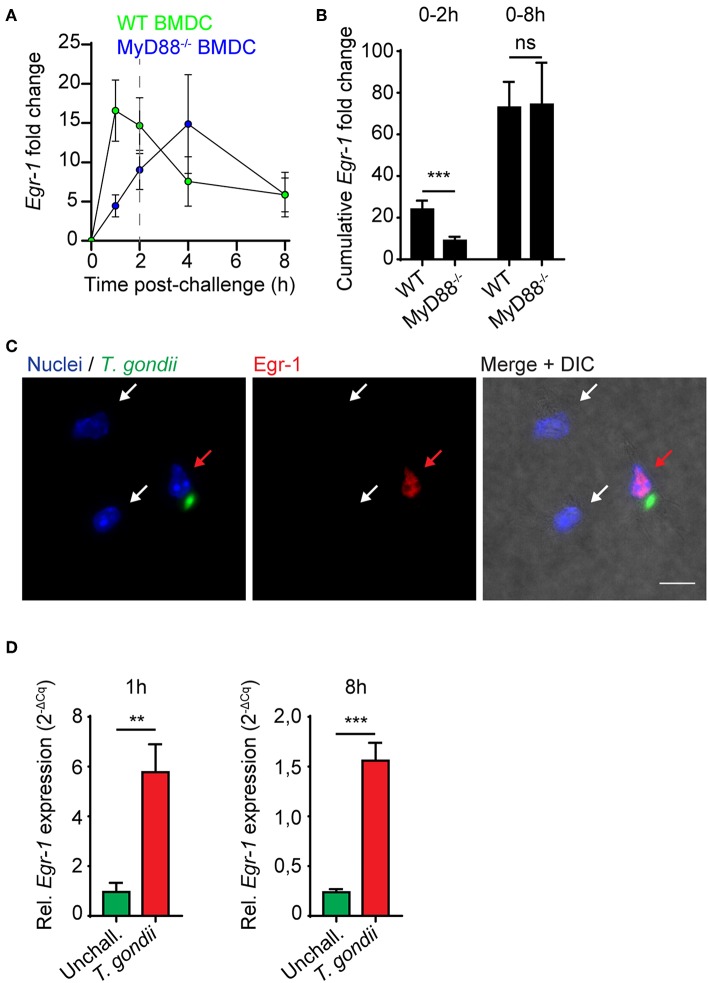
*Egr-1* mRNA expression in MyD88-deficient murine BMDCs and in human MoDCs. **(A)** qPCR analysis of Egr-1 cDNA from MyD88^−/−^ or wild type (WT) BMDCs challenged with freshly egressed *T. gondii* tachyzoites (PTG) related to unchallenged BMDCs in complete medium. Each timepoint represents the mean ± SEM from 3 independent experiments. **(B)** Cumulative Egr-1 expression, as in **(A)**, calculated by area under the curve analysis (***p ≤ 0.001, ns *p* > 0.05, permutation test). **(C)** Representative micrographs of MyD88^−/−^ BMDCs challenged with freshly egressed *T. gondii* tachyzoites (PTG; green) for 8 h, stained for Egr-1 (red), and with DAPI (blue). Red arrow indicates infected DC. White arrows indicate by-stander DCs. Scale bar 10 μm. **(D)** qPCR analysis of Egr-1 cDNA from unchallenged MoDCs or MoDCs challenged with freshly egressed *T. gondii* tachyzoites (PRUku80) at indicated timepoints. Relative expression (2^−ΔCq^) is displayed as mean ± SE (*n* = 6–7, **p ≤ 0.01, ***p ≤ 0.001, Mann-Whitney).

Toxoplasma antigens function as ligands for multiple TLRs, with important roles attributed to TLR11 and TLR12 in mice (Yarovinsky et al., 2005; Koblansky et al., 2013). Cytokine responses by DCs in response to soluble tachyzoite antigen are highly dependent on the TLR-associated adapter protein MyD88 (Scanga et al., 2002). Immediate-early *Egr-1* induction by the TLR4-ligand LPS has been previously reported in murine peritoneal macrophages (Coleman et al., 1992). We therefore considered that the *Egr-1* mRNA induction in response to LPS, heat-inactivated tachyzoites, tachyzoite lysate and live tachyzoites observed here could be mediated by TLR/MyD88-dependent signaling. Importantly, in MyD88-deficient BMDCs, the immediate-early *Egr-1* mRNA up-regulation was substantially reduced while the extended *Egr-1* mRNA up-regulation phase was retained (Figure 2A). Importantly, while the cumulative *Egr-1* response was significantly reduced at the immediate-early phase, non-significant differences were observed at 8 h (Figure 2B). Similar to wild-type BMDCs (Figure 1E), nuclear translocation of Egr-1 was observed in MyD88-deficient BMDCs upon infection with *T. gondii* tachyzoites (Figure 2C). Additionally, human monocyte-derived DCs (MoDCs) exhibited a notable induction of *Egr-1* mRNA both at 1 and 8 h post-challenge with tachyzoites, similar to murine BMDCs (Figure 2D). As the human genome lacks functional expression of TLR11/12 (Yarovinsky et al., 2005; Koblansky et al., 2013), the data exclude a critical dependency on TLR11/12 for the immediate-early and the extended phase Egr-1 responses. Jointly, the data exclude a critical role for MyD88/TLR signaling for the extended-phase Egr-1 response of parasitized DCs.

### The Immediate-Early *Egr-1* Response of *T. gondii*-challenged Murine and Human DCs Depends Primarily on the ERK1/2 MAPK Signaling Pathway

**Figure 3:**
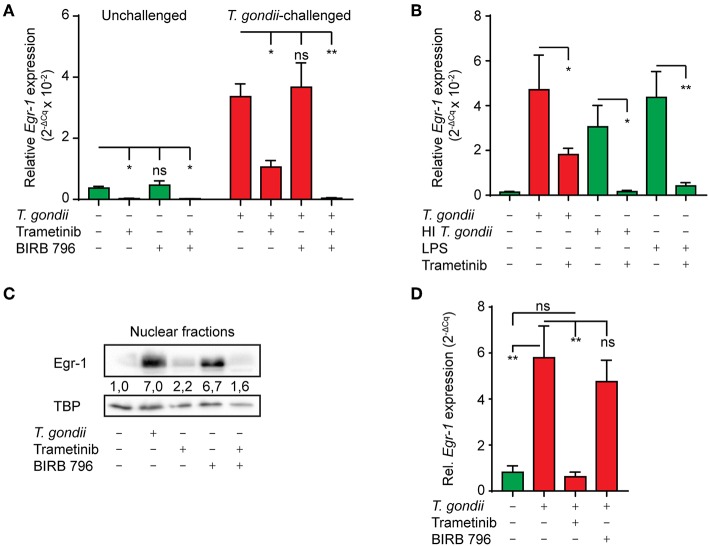
Impact of ERK1/2-pathway inhibition on the immediate-early *Egr-1* mRNA expression of BMDCs and MoDCs challenged with *T. gondii*. **(A)** qPCR analysis of Egr-1 cDNA from unchallenged BMDCs or BMDCs challenged with freshly egressed *T. gondii* tachyzoites (PRUku80, MOI 3) and treated with MEK1/2 inhibitor (Trametinib) and/or pan-p38 inhibitor (BIRB 796) for 1 h. Relative expression (2^−ΔCq^) is displayed as mean ± SE (*n* = 5 for unchallenged, *n* = 3 for challenged; *p ≤ 0.05, **p ≤ 0.01, ns *p* > 0.05, ANOVA, Dunnett's). **(B)** qPCR analysis of Egr-1 cDNA from BMDCs challenged with freshly egressed *T. gondii* tachyzoites (PRUku80 MOI 3), LPS 10 ng/mL or heat-inactivated (HI) tachyzoites (PRUku80 MOI 3 equivalent), or left unchallenged BMDCs for 1 h in complete medium (CM). Relative expression (2^−ΔCq^) is displayed as mean ± SE (*n* = 3; *p ≤ 0.05, **p ≤ 0.01, ANOVA, Tukey HSD). **(C)** Western blot with densitometric analysis of nucleus-enriched (nuclear) fractions of BMDCs challenged with freshly egressed *T. gondii* tachyzoites (PRUku80) and treated with Trametinib and/or BIRB 796 for 2 h or left unchallenged in CM and probed for Egr-1 or TATA-binding protein (TBP). Representative of 3 independent experiments. **(D)** qPCR analysis of Egr-1 cDNA from unchallenged MoDCs or MoDCs challenged with freshly egressed *T. gondii* tachyzoites (PRUku80) and treated with Trametinib or BIRB 796 as in **(A)**. Relative expression (2^−ΔCq^) is displayed as mean ± SE (*n* = 4; **p ≤ 0.01, *p ≤ 0.05, ANOVA, Tukey HSD).

Challenge of murine macrophages with *T. gondii* activates the extracellular signal-regulated kinase 1/2 (ERK1/2) and p38 MAPK pathways (Kim et al., 2004). It is well-established that activation of these pathways can result in transcriptional activation of *Egr-1* (Lim et al., 1998; Guha et al., 2001). To determine the contribution of ERK1/2 and p38 MAPK to Egr-1 responses, we made use of Trametinib, a selective allosteric inhibitor of the ERK1/2-activating kinase MEK1/2, and the selective allosteric pan-p38 MAPK inhibitor BIRB 796 (Pargellis et al., 2002; Gilmartin et al., 2011), respectively. Inhibition of MEK1/2 (Trametinib) nearly abolished *Egr-1* mRNA expression (93 (± 3) % reduction) in unchallenged BMDCs and significantly reduced the *Egr-1* mRNA induction in *T. gondii*-challenged BMDCs 1 h after challenge (Figure 3A). In contrast, pan-p38 MAPK inhibitor (BIRB796) treatment non-significantly affected *Egr-1* mRNA expression in unchallenged and *T. gondii*-challenged BMDCs (Figure 3A). However, combined p38 MAPK and MEK1/2 inhibition abolished the *Egr-1* mRNA up-regulation in *T. gondii*-challenged BMDCs 1 h post-challenge and significantly reduced *Egr-1* mRNA expression (99 (± 1) % reduction). Further, MEK1/2 inhibition nearly abolished Egr-1 responses in BMDCs challenged with heat-inactivated *T. gondii* or LPS (Figure 3B). Western blot analyses corroborated a reduced abundancy of Egr-1 protein in nucleus-enriched fractions upon MEK1/2 inhibition (Figure 3C). Similarly, in human MoDCs, MEK1/2 inhibition abolished the *Egr-1* mRNA induction after *T. gondii-*challenge (Figure 3D).

The data indicate that the immediate-early Egr-1 response of DCs to live tachyzoites is primarily dependent on ERK1/2 signaling, with a minor contribution of the p38 MAPK pathway.

### The Extended Phase *Egr-1* Response in *T. gondii*-challenged Murine and Human DCs Depends on p38 MAPK Signaling

**Figure 4:**
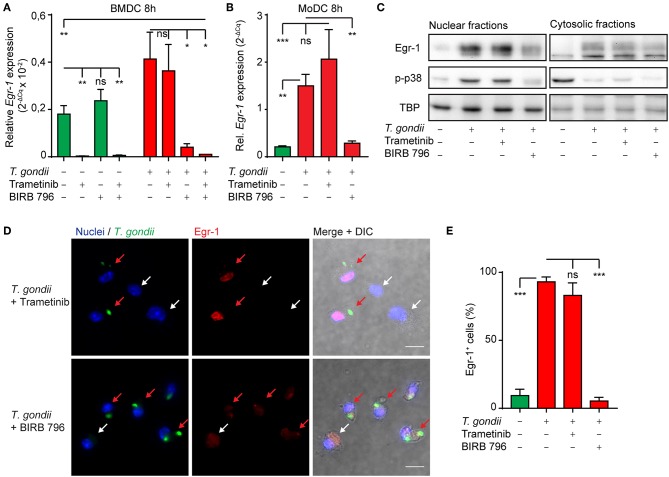
Impact of p38 MAPK inhibition on the extended phase Egr-1 expression of BMDCs and MoDCs challenged with *T. gondii*. **(A)** qPCR analysis of Egr-1 cDNA from BMDCs unchallenged or challenged with freshly egressed *T. gondii* tachyzoites (PRUku80) and/or treated with MEK1/2 (Trametinib) and/or pan-p38 inhibitors (BIRB-796) for 8 h in complete medium (*n* = 3, *p ≤ 0.05, ns *p* > 0.05, ANOVA, Dunnett's). **(B)** qPCR analysis of Egr-1 cDNA from MoDCs treated as in **(A)** (*n* = 4–7, *p ≤ 0.05, **p ≤ 0.01, ***p ≤ 0.001, Kruskal-Wallis, Dunn's). **(C)** Representative Western blot of BMDCs challenged with freshly egressed *T. gondii* tachyzoites (PRUku80) for 8 h and treated with Trametinib/BIRB-796) or unchallenged and probed for Egr-1, phospho-p38 (Thr180/Tyr182), or TATA-binding protein (TBP). Representative of 3 experiments. **(D)** Representative micrographs of BMDCs challenged with freshly egressed GFP-expressing *T. gondii* tachyzoites (PTG) and treated with Trametinib or BIRB 796 for 8 h. Cells stained for Egr-1 (red) and with DAPI (blue). Red arrows indicate infected DCs (green). White arrows indicate by-stander DCs. Scale bar 10 μm. **(E)** Frequency of detectable Egr-1 expression related to the total population in BMDCs infected with *T. gondii* tachyzoites (PTG) and treated with Trametinib or BIRB 796 for 8 h. Relative expression was determined by pixel threshold analysis as indicated under Materials and Methods. Bars show the mean ± SEM from 3 independent experiments (*n* = 3, ***p ≤ 0.001, ns *p* > 0.05, ANOVA, Tukey HSD).

Given the dependency of the early-immediate Egr-1 response on ERK1/2 signaling, we sought to determine MAPK signaling during the extended phase Egr-1 response. Interestingly, MEK1/2 inhibition (Trametinib) strongly reduced baseline *Egr-1* mRNA expression in unchallenged BMDC, but non-significantly inhibited *Egr-1* expression in *T. gondii*-challenged BMDCs (Figure 4A). In contrast, treatment with the pan-p38 MAPK inhibitor (BIRB796) non-significantly affected *Egr-1* expression in unchallenged BMDCs, while the *Egr-1* mRNA induction in *T. gondii*-challenged DCs was nearly abolished (Figure 4A). Additionally, the *Egr-1* expression was reduced well below baseline expression level with combined MEK1/2 and pan-p38 MAPK inhibition 8 h post-challenge (Figure 4A). These dramatic differences were confirmed in human MoDCs. Pan-p38 inhibition abolished the *Egr-1* mRNA upregulation 8 h after challenge with *T. gondii* tachyzoites while MEK1/2 inhibition non-significantly elevated *Egr-1* expression (Figure 4B). Western blotting analyses corroborated that pan-p38 MAPK inhibition, but not MEK1/2, reduced signal corresponding to Egr-1 protein (Figure 4C). Similarly, immunofluorescence microscopy confirmed that the localization of Egr-1 protein to the cell nucleus was abrogated by pan-p38 MAPK inhibition and non-significantly affected by MEK1/2 inhibition (Figures 4D,E). We conclude that the extended phase *Egr-1* mRNA induction and the elevated nuclear Egr-1 expression in *T. gondii*-challenged DCs are p38 MAPK-dependent.

### The *T. gondii* Dense Granule Protein GRA24 Impacts on the Extended Phase *Egr-1* Response

**Figure 5:**
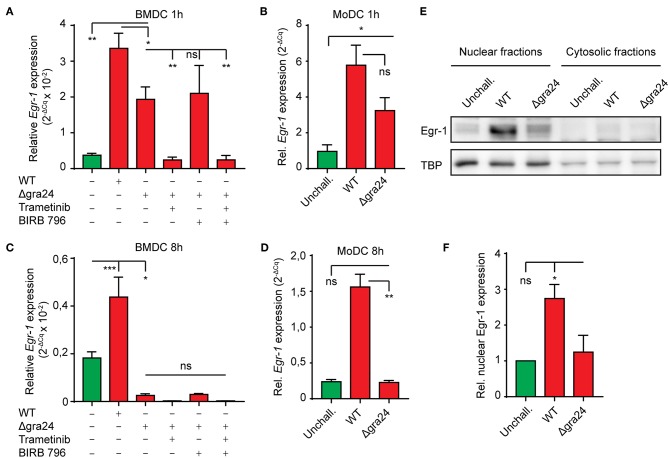
Role of the parasite-derived secreted effector GRA24 on the extended phase Egr-1 expression. **(A)** qPCR analysis of Egr-1 cDNA from BMDCs challenged with freshly egressed Δgra24 or WT *T. gondii* tachyzoites (PRUku80) and treated with MEK1/2 inhibitor (Trametinib) and/or pan-p38 inhibitor (BIRB 796) for 1 h in complete medium. Relative expression (2^−ΔCq^) is displayed as mean ± SE (*n* = 3–5, **p ≤ 0.01, *p ≤ 0.05, ns *p* > 0.05, ANOVA, Dunnett's). **(B)** qPCR analysis of Egr-1 cDNA from MoDCs challenged with freshly egressed Δgra24 or WT *T. gondii* tachyzoites (PRUku80) 1 h in complete medium. Relative expression (2^−ΔCq^) is displayed as mean ± SE (*n* = 6–7, *p ≤ 0.05, ns *p* > 0.05, Kruskal-Wallis, Dunn's). **(C)** qPCR analysis of Egr-1 cDNA from BMDCs challenged with freshly egressed Δgra24 or WT *T. gondii* tachyzoites (PRUku80) and treated with Trametinib and/or BIRB 796 for 8 h in complete medium. Relative expression (2^−ΔCq^) is displayed as mean ± SE (*n* = 4–5, ***p ≤ 0.001, *p ≤ 0.05, ns *p* > 0.05, ANOVA, Tukey HSD). **(D)** qPCR analysis of Egr-1 cDNA from MoDCs challenged with freshly egressed Δgra24 or WT *T. gondii* tachyzoites (PRUku80) for 8 h in complete medium. Relative expression (2^−ΔCq^) is displayed as mean ± SE (*n* = 6–7, **p ≤ 0.01, ns *p* > 0.05, Kruskal-Wallis, Dunn's). **(E)** Representative Western blot of BMDCs challenged with freshly egressed Δ gra24 or WT *T. gondii* tachyzoites (PRUku80) for 8 h in complete medium and separated into nucleus- (nuclear) and cytosol-enriched (cytosolic) fractions and probed for Egr-1 or TATA-binding protein (TBP). **(F)** Densitometric analysis of Western blots, as in **(E)** displayed as the mean ± SEM from 3 independent experiments (*p ≤ 0 05, ns *p* > 0.05, ANOVA, Dunnett's).

Upon invasion of the host cell, the *T. gondii* dense granule protein GRA24 is secreted in the host cell cytosol and complexes with host cell p38α in fibroblast and J774 macrophage cell lines (Braun et al., 2013). This interaction results in sustained autophosphorylation of host p38α and prolonged up-regulation of immediate-early genes, including *Egr-1*. Given the dependency of the extend phase *Egr-1* upregulation on p38 MAPK, we sought to confirm its dependency on GRA24 in primary DCs (Braun et al., 2013). To this end we compared *Egr-1* expression in BMDCs challenged with wild type (WT) or GRA24-deficient (Δgra24) PRUku80 tachyzoites. One h post-challenge, both murine BMDCs and human MoDCs challenged with Δgra24 tachyzoites exhibited a robust *Egr-1* mRNA induction, yet significantly smaller than the induction in WT tachyzoite-challenged BMDCs (Figures 5A,B). However, the *Egr-1* expression was reduced below baseline by MEK1/2 inhibition (Figure 5A). In stark contrast, at 8 h post-challenge, BMDCs challenged with Δgra24 tachyzoites exhibited strongly down-regulated *Egr-1* mRNA expression (Figure 5C) and MoDCs exhibited non-significant differences in Egr-1 expression compared with unchallenged MoDCs (Figure 5D). Western blotting showed reduced Egr-1 signal upon challenge with Δgra24 tachyzoites, indicating that nuclear Egr-1 levels in *T. gondii*-challenged BMDCs at 8 h post-challenge are to a high extent GRA24-dependent (Figures 5E,F). Thus, while the immediate-early Egr-1 induction was not critically dependent on GRA24, the extended phase Egr-1 response was GRA24-dependent in both MoDCs and BMDCs.

### Hypermotility of *T. gondii*-infected BMDCs Depends on MAPK Signaling but Not on *Egr-1* Expression

**Figure 6:**
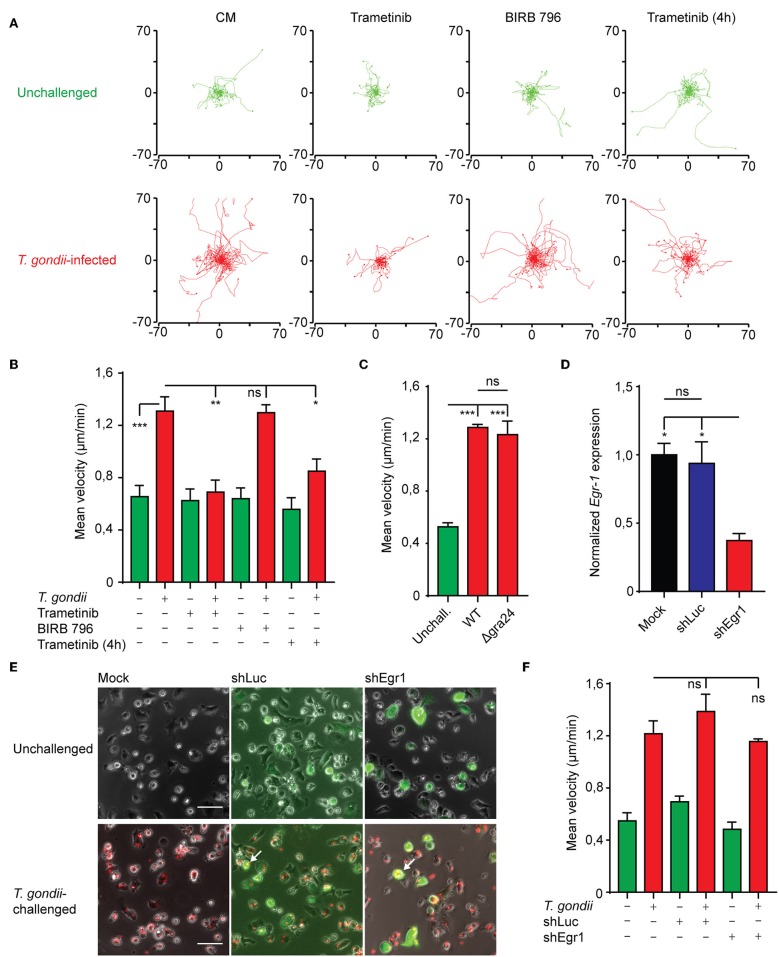
Impact of MAPK inhibition on BMDC motility and motility analyses of *Egr-1*-silenced BMDCs. **(A)** Representative motility plots of BMDCs infected with freshly egressed *T. gondii* tachyzoites (PRU-RFP MOI 3) for 4 h in the presence of Trametinib or BIRB 796 for 4 h or with Trametinib added directly before a 1 h motility assay. **(B)** Quantification of the velocity of BMDCs from 4 independent experiments as in **(A)** (*p ≤ 0.05, **p ≤ 0.01, ***p ≤ 0.001, ANOVA, Tukey HSD). **(C)** Quantification of the velocity of BMDCs infected with freshly egressed Δgra24 or WT *T. gondii* tachyzoites (PRUku80) or unchallenged for 4 h in a 1 h motility assay (*n* = 3, ***p ≤ 0.001, ns > 0,05, ANOVA, Tukey HSD). **(D)** qPCR analysis of Egr-1 cDNA from mock transduced BMDCs or BMDCs transduced with shLuc-GFP or shEgr1-GFP lentivirus. Expression is normalized to mock and displayed as mean ± SE (*n* = 3, *p ≤ 0.05, ns *p* > 0.05, ANOVA, Tukey HSD). **(E)** Representative micrographs of mock transduced BMDCs or BMDCs transduced with shLuc-GFP or shEgr1-GFP lentivirus either left unchallenged or challenged with *T. gondii* tachyzoites (PRU-RFP MOI 3) for 4 h. White arrows indicate infected (RFP^+^) and transduced (GFP^+^) DCs. Scale bar 50 μm. **(F)** Quantification of the velocity of mock transduced BMDCs or BMDCs transduced with shLuc-GFP or shEgr1-GFP lentivirus either left unchallenged or challenged with *T. gondii* tachyzoites (PRU-RFP MOI 3) for 4 h in a 1 h motility assay (*n* = 3, ns *p* > 0.05, ANOVA, Tukey HSD).

Cell motility is intimately linked with MAPK signaling and Egr-1 has also been linked to cell migration in non-hematopoietic cells (Sarver et al., 2010; Tarcic et al., 2012). To determine whether MAPK signaling and Egr-1 expression contributed to previously characterized migratory responses upon *T. gondii* infection (Fuks et al., 2012; Kanatani et al., 2017), we first applied pharmacological inhibition of MEK1/2 and p38 MAPK. BMDCs infected with *T. gondii* migrated longer distances and displayed significantly higher velocities compared with unchallenged BMDCs (Figures 6A,B), in line with the induction of hypermotility in DCs upon *T. gondii* invasion (Lambert et al., 2006; Weidner et al., 2013; Kanatani et al., 2017). Interestingly, p38 MAPK inhibition non-significantly affected the migrated distances and velocity of infected BMDCs, indicating that p38 does not impact on the migratory functions of DCs. In stark contrast, inhibition of MEK1/2 essentially abolished *T. gondii*-induced hypermotility (Figures 6A,B). Both inhibitors non-significantly affected the baseline motility of unchallenged BMDCs. Additionally, MEK1/2 inhibition well beyond the ERK-mediated immediate-early phase of *Egr-1* induction (4 h) significantly reduced hypermotility of *T. gondii*-infected BMDCs (Figures 6A,B), indicating implication of ERK1/2 MAPK signaling on migratory responses at later timepoints. Further, WT and Δgra24 parasites similarly induced hypermotility in DCs (Figure 6C).

To more specifically asses the role of Egr-1 in hypermotility, we employed a lentivirus-mediated shRNA approach targeting the *Egr-1* transcript (shEgr1). *Egr-1*-silenced BMDCs expressed significantly lower levels of *Egr-1* mRNA than BMDCs transduced with a control lentivirus (shLuc) or mock transduction (Figure 6D). Next, we assessed the motility and velocities of *Egr-1*-silenced DCs (GFP^+^). *T. gondii*-infected *Egr-1*-silenced BMDCs (GFP^+^RFP^+^) displayed migratory velocities comparable to those of control- and mock-transduced cells (Figures 6E,F). We conclude that Egr-1 expression and p38 MAPK signaling non-significantly impact on Toxoplasma-induced hypermigration of BMDCs.

### GRA24 Impacts on Transcriptional IL-12 and IL-2 Responses in *T. gondii*-challenged BMDCs

**Figure 7:**
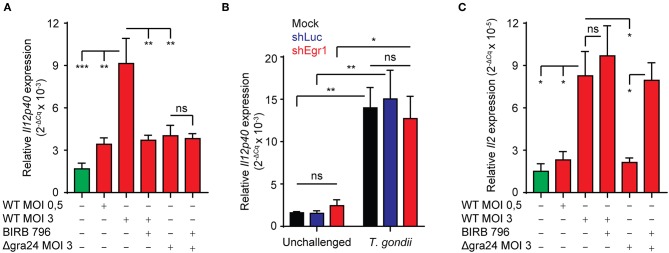
Role of GRA24 and Egr-1 in *T. gondii*-induced IL-12p40 and IL-2 mRNA expression. **(A)** qPCR analysis of Il12p40 cDNA from BMDCs challenged for 8 h with freshly egressed Δgra24 or WT *T. gondii* tachyzoites (PRUku80) at indicated MOI and treated with pan-p38 inhibitor (BIRB 796) or left untreated. Relative expression (2^−ΔCq^) is displayed as mean ± SE (*n* = 4, **p ≤ 0.01, ***p ≤ 0.001, ns *p* > 0.05, ANOVA, Tukey HSD). **(B)** qPCR analysis of Il12p40 cDNA from mock transduced BMDCs or BMDCs transduced with shLuc-GFP or shEgr1-GFP lentivirus and challenged for 8 h with freshly egressed *T. gondii* tachyzoites (PRU-RFP, MOI 3). Relative expression (2^−ΔCq^) is displayed as mean ± SE (*n* = 5, **p ≤ 0.01, *p ≤ 0.05, ns *p* > 0.05, ANOVA, Tukey HSD). **(C)** qPCR analysis of Il2 cDNA from BMDCs challenged for 8 h with freshly egressed Δgra24 or WT *T. gondii* tachyzoites (PRUku80) at indicated MOI and treated with pan-p38 inhibitor (BIRB 796) or left untreated. Relative expression (2^−ΔCq^) is displayed as mean ± SE (*n* = 4, *p ≤ 0.05, ns *p* > 0.05, ANOVA, Tukey HSD).

IL-12p40 responses in macrophages have been shown to depend on GRA24/p38 signaling (Kim et al., 2005; Braun et al., 2013). Also, DCs are early producers of IL-2 and their ability to elicit IFNγ expression in NK cells is dependent on IL-2 (Granucci et al., 2004). We thus sought to determine whether BMDCs upregulate IL-2 and IL-12p40 expression upon *T. gondii*-challenge. We found that IL-12p40 mRNA expression was induced in a MOI-dependent fashion and that this induction was significantly reduced in presence of pan-p38 MAPK inhibitor. Importantly, the observed induction was significantly less prominent upon challenge with GRA24-deficient (Δgra24) *T. gondii* (Figure 7A). In contrast, Egr-1 silencing had a non-significant impact on IL-12p40 mRNA expression (Figure 7B). Also, a significantly stronger induction of IL-12p40 mRNA was observed upon challenge with type II strains compared to type I (Figure S3). Further, IL-2 expression was also upregulated in BMDCs challenged with tachyzoites in a MOI-dependent manner (Figure 7C). Unlike IL-12 response, this upregulation was non-significantly affected by pan-p38 MAPK inhibition. IL-2 expression was significantly lower in BMDCs challenged with GRA24-deficient (Δgra24) compared to WT *T. gondii* (PRUku80) and increased by pan-p38 inhibition. Altogether, the data indicate that transcriptional IL-12 responses are mediated in a GRA24/p38-dependent, but Egr-1-independent, fashion in Toxoplasma-challenged BMDCs and that IL-2 mRNA responses are mediated in a GRA24-dependent but p38-independent manner.

### *Egr-1* Expression Impacts on BMDC Maturation

**Figure 8:**
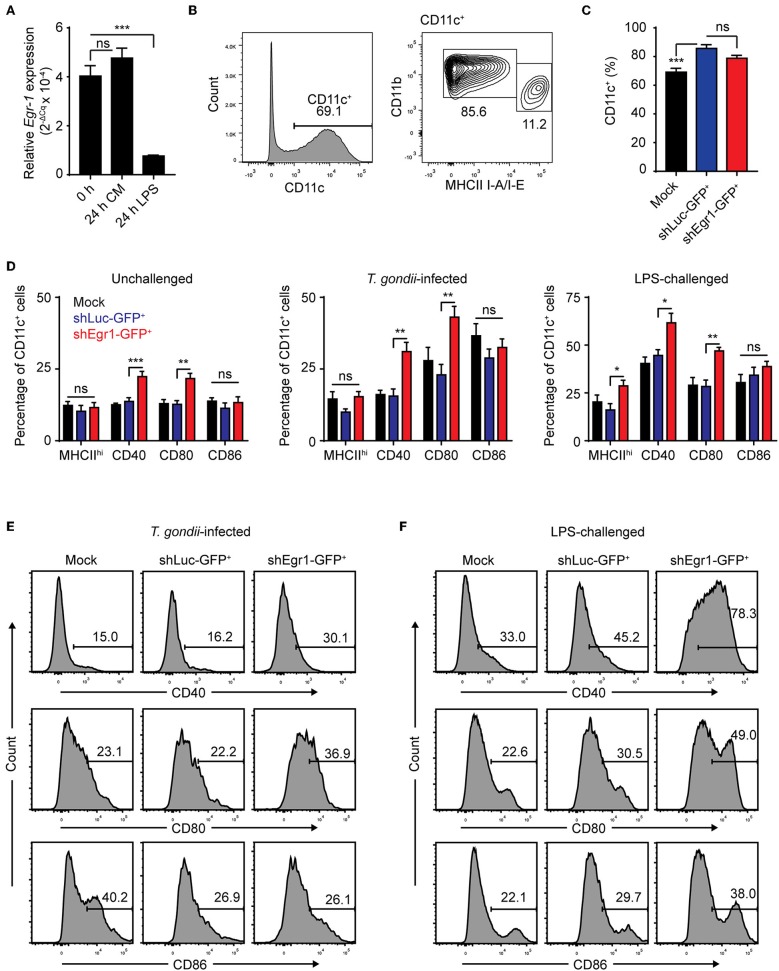
Effects of *Egr-1* silencing on the phenotypic maturation of BMDCs upon challenge with *T. gondii* and LPS. **(A)** qPCR analysis of Egr-1 cDNA from BMDCs at day 8 of GM-CSF culture (0 h) and challenged with 100 ng/mL LPS for 24 h or after continued culture for 24 h (24 h CM). Relative expression (2^−ΔCq^) is displayed as mean ± SE (*n* = 3, ***p ≤ 0.001, ns *p* > 0.05, ANOVA, Dunnett's). **(B)** Representative phenotype of loosely adherent mock transduced cells in GM-CSF BM cultures at day 9 showing CD11b^hi^MHCII^low^ and CD11b^int^MHCII^hi^ BMDCs. **(C)** Flow cytometric analysis of CD11c expression on mock transduced and GFP^+^ shLuc- or shEgr1-transduced BMDCs gated as in **(B)** (*n* = 6, ***p ≤ 0.001, ns *p* > 0.05, ANOVA, Tukey HSD). **(D)** Flow cytometric analysis as in **(B)** of CD11b^int^MHCII^hi^ (MHCII^hi^), CD40, CD80, and CD86 expression on mock transduced and GFP^+^ shLuc- or shEgr1-transduced CD11c^+^ BMDCs that were challenged with *T. gondii* tachyzoites (PRU-RFP MOI 1) or 100 ng/mL LPS or left unchallenged and cultured for 24 h. Displayed is the mean percentage of positive cells (± SE) of 6 independent samples (*n* = 6, *p ≤ 0.05, **p ≤ 0.01, ***p ≤ 0.001, ns *p* > 0.05, ANOVA, Tukey HSD). **(E)** Representative density plots of a single sample of CD40, CD80 and CD86 expression as in **(D)** of mock transduced and GFP^+^ shLuc- or shEgr1-transduced BMDCs infected with *T. gondii* tachyzoites for 24 h. **(F)** Representative density plots of a single sample of CD40, CD80, and CD86 expression as in **(D)** of mock transduced and GFP^+^ shLuc- or shEgr1-transduced BMDCs challenged with 100 ng/mL LPS for 24 h.

We have previously shown that *T. gondii*-challenge induces elevation of maturation markers in BMDCs and MoDCs, albeit to a lesser extent than LPS (Lambert et al., 2006; Fuks et al., 2012). Egr-1 has been pegged as a factor that promotes differentiation along the monocytic lineage at the expense of the granulocytic lineage and expression of Egr-1 increases with murine DC development (Krishnaraju et al., 2001; Lin et al., 2015). Along these lines, a 24 h challenge with LPS led to a significant down-regulation of *Egr-1* expression, unlike continued culture (Figures 1A, 8A). To determine the role of Egr-1 in BMDC differentiation and maturation, we first assessed a number of phenotypical markers in *Egr-1*-silenced BMDCs (shEgr1) by flow cytometry (Figures S4A,B). Even though we could confirm a significantly higher mRNA expression of neutrophil elastase in shEgr1-transduced BMDCs (Figure S4C), irrespective of transduction condition, BMDC cultures yielded a loosely-adherent population that was predominantly composed of CD11c^+^ cells (Figures 8B,C). The significantly increased fraction of CD11c^+^ cells among shLuc-GFP^+^ cells we attribute to lentiviral transduction. The CD11c^+^ population had a fraction with a CD11b^hi^MHCII^low^ (MHCII^low^) phenotype and a smaller fraction of CD11b^int^MHCII^hi^ cells (MHCII^hi^). Concluding that Egr-1 silencing did not impair differentiation of either subset, we next assessed the impact of Egr-1 silencing on BMDC phenotypic maturation. Silencing of *Egr-1* non-significantly impacted on the ratio of MHCII^low^/MHCII^hi^ BMDCs compared with control transduction (Figure 8D). For all treatments, the Egr-1-silenced CD11c^+^ BMDC population was significantly more often positive for CD40 and CD80 than its mock- and shLuc-transduced counterparts (Figure 8D). Importantly, for both *T. gondii*-infected and LPS-treated BMDCs, Egr-1 silencing led to a significant elevation of maturation markers CD40 and CD80 (Figures 8E,F, Figure S4D). Altogether, this indicates that Egr-1 acts as a negative regulator of phenotypic DC maturation upon *T. gondii* infection and LPS-treatment.

## Discussion

Because Egr-1 is an important mediator and regulator of inflammatory responses to extracellular stimuli, we investigated whether intracellular signaling in an infection model could also mediate Egr-1 responses. We report that intracellular *T. gondii* tachyzoites substantially modulate the phenotypical maturation of DCs via Egr-1 and MAP kinase signaling, with maintained migratory responses.

We demonstrate that the expression of the transcription factor and inflammatory regulator Egr-1 is significantly modulated in DCs by *T. gondii* infection in a biphasic fashion. Previous studies described induction of Egr-1 and−2 transcription upon *T. gondii* challenge in fibroblasts and cell lines (Phelps et al., 2008; Wiley et al., 2011). Here, we analyzed Egr-1 responses over time in primary human and murine DCs and report that heat-inactivated tachyzoites and tachyzoite lysate induced an immediate-early Egr-1 response, similar to LPS (Guha et al., 2001). In stark contrast, live tachyzoites additionally induced maintained Egr-1 expression during an extended phase. Importantly, the elevated Egr-1 expression localized to nucleus-enriched fractions of Toxoplasma-challenged DCs. Moreover, immunofluorescence analyses showed abundant nuclear Egr-1 signal in Toxoplasma-infected DCs but not in by-stander DCs or in unchallenged DCs. Jointly, this indicated that elevated amounts of Egr-1 were translocated to the cell nucleus in Toxoplasma-challenged DCs and that this response was associated to the presence of live intracellular tachyzoites in DCs. Soluble microbial antigens, live bacteria and other inflammatory stimuli have been shown to induce Egr-1 responses (de Grado et al., 2001; Bhattacharyya et al., 2013; de Klerk et al., 2017). Similarly, our data show that *T. gondii*-derived soluble fractions can generate immediate-early Egr-1 responses. Additionally, we show that intracellular live *T. gondii* induce extended/sustained Egr-1 (and Egr-2) responses in DCs, in line with responses in macrophages (Braun et al., 2013). This motivated a further exploration of the mechanisms of activation and the putative impact on DC function.

**Figure 9:**
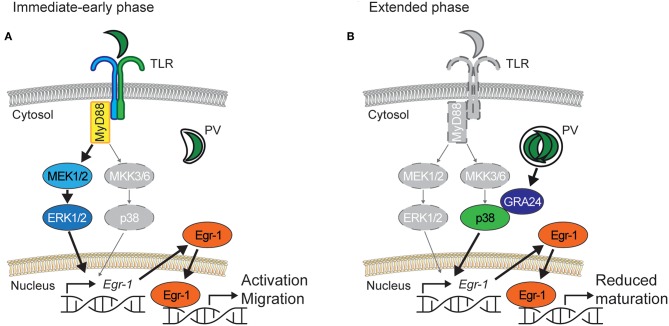
Proposed bi-phasic model for the role of Egr-1 in activation of DCs and down-modulation of DC maturation upon challenge with *T. gondii*. **(A)** Immediate-early phase. Similar to LPS, parasite antigens contained in lysate, or heat-inactivated parasites activate TLRs that signal through the adaptor MyD88 and the ERK1/2 MAPK signaling pathway to induce rapid Egr-1 expression. MyD88-deficient DCs exhibit a strongly delayed Egr-1 response and MEK1/2 inhibition abolishes Egr-1 activation, with a minor contribution of the p38 MAPK pathway. Rapid high Egr-1 expression is consistent with cell activation and migration. The migratory responses of *T. gondii*-challenged DCs are abolished by inhibition of ERK1/2 signaling. **(B)** Extended phase. In actively invaded DCs, intracellular *T. gondii* tachyzoites replicate in a parasitophorous vacuole (PV) and secrete in the host DC cytosol the p38-interacting protein GRA24, leading to p38 phosphorylation. Pharmacological inhibition of p38 phosphorylation or challenge with GRA24-deficient *T. gondii* abolish a sustained Egr-1 response. Sustained Egr-1 expression leads to a down-modulation of DC maturation. In *Egr-1*-silenced DCs maturation is elevated upon LPS or *T. gondii* challenge. For **(A,B)**, vivid colored items indicate the predominantly active signaling pathway and gray-colored items indicate the less active signaling pathway.

Our data show that Egr-1 expression can be induced in primary DCs by both canonical and non-canonical pathways. Egr-1 can be induced by a number of endogenous and exogenous stimuli (Bhattacharyya et al., 2013). Egr-1 expression has also been recently linked to MyD88/TLR activation (Famakin et al., 2014) and TLRs, especially TLR11/12 have been reported to mediate important responses for the mitigation of *T. gondii* infection (Yarovinsky et al., 2005; Koblansky et al., 2013). Our data show that, on one hand, soluble parasite-derived fractions induce *canonical* immediate-early Egr-1 responses and, on the other hand, intracellular localization of live tachyzoites also induces a *non-canonical* sustained Egr-1 expression (Figure 9). Further, antigens from intracellular parasites may be transported to the host cell cytosol and even secreted from infected cells, which may drive responses through canonical pattern-recognition receptors (PRR)/TLR pathways (Blanchard et al., 2008; Braun et al., 2013). To this end, we assessed responses in MyD88^−/−^ BMDCs, and in human MoDCs, which lack functional TLR11/12. In MyD88^−/−^ BMDCs, the onset of the Egr-1 response was delayed (immediate-early phase), while a similar cumulative Egr-1 activation was observed over time. Similar to murine BMDCs, human MoDCs responded with Egr-1 expression at the early-immediate and extended phases upon challenge with *T. gondii*. Jointly, this indicated that MyD88- and TLR11/12-signaling were not essential for the extended phase Egr-1 responses. Yet, alternative signaling which could mediate the responses include MyD88-independent TLR signaling and alternative PRRs. However, these findings motivated an assessment of MAPK signaling presumably upstream of the Egr-1 response.

We show that different MAPK pathways preferentially mediate the immediate-early and extended phase Egr-1 responses. While the immediate-early response was dependent on ERK1/2 MAPK signaling, the extended phase Egr-1 response depended chiefly on p38 MAPK signaling. This was corroborated using parasites deficient in the p38-interacting GRA24 protein (Braun et al., 2013), which yielded dramatically reduced Egr-1 responses in the extended phase while the immediate-early response was maintained. Multiple reports indicate that the immediate-early Egr-1 responses are mediated by ERK1/2 but not p38 (Guha et al., 2001; Sobke et al., 2006; de Klerk et al., 2017). However, a contribution of p38-mediated signaling has also been reported in some bacterial, viral and toxicity models (Kenzel et al., 2009; Li et al., 2016; Kim et al., 2019). Our data shows that Egr-1 responses can selectively be mediated through p38 or ERK1/2 MAPK signaling by different stimuli. Because the parasite-derived secreted protein GRA24 has been shown to modulate cytokine production in macrophages via p38 signaling (Braun et al., 2013), we asked whether the observed p38-related Egr-1 responses impacted on DC functions.

We report that Egr-1 responses impact on the maturation of immature DCs. *T. gondii* infection leads to moderate elevation of maturation markers in MoDCs and BMDCs, albeit significantly inferior to stimulation with LPS (Lambert et al., 2006; Fuks et al., 2012). In line with this, active invasion by Toxoplasma blocked LPS-induced maturation of BMDCs and their capacity to activate CD4^+^ T cells (McKee et al., 2004). Interestingly, gene silencing of Egr-1 in BMDCs led to elevated expression of maturation markers upon challenge with *T. gondii* or LPS, indicating a link to Egr-1 expression but not specifically to *T. gondii* infection. Egr-1 impacts directly and indirectly on a number of signaling pathways implicated in the transcriptional regulation of inflammation and the cellular immune response. How the downmodulation of co-stimulatory molecules is effectuated awaits further investigation. The data suggest nevertheless that Egr-1 can act as a negative regulator of DC maturation. In line with this, the related transcription factor Egr-2 has been attributed functions as a negative regulator of DC immunogenicity (Miah et al., 2013). We observed a similar biphasic and sustained expression pattern for Egr-2 and Egr-1, indicating that Egr-2 may additionally contribute to down-modulation of DC maturation. Further, the migratory responses of DCs to *T. gondii* challenge were abolished by ERK1/2 inhibition but non-significantly modulated by Egr-1 silencing. This would suggest that, in parasitized DCs, migratory responses are maintained while activation is suppressed or down-modulated (Figure 9). In line with this, recent reports show that Egr-1 responses promote epithelial-mesenchymal transition (EMT) and metastasis in malignancies (Shao et al., 2018; Wang et al., 2019). *T. gondii* encounters DCs in peripheral tissues, such as the intestine, and DCs function as shuttles (“Trojan horse”) that potentiate the systemic dissemination of the parasite (Courret et al., 2006; Lambert et al., 2006; Bierly et al., 2008). Recent evidence shows that *T. gondii* modulates the motility of parasitized DCs and other immune cells (Olafsson et al., 2018, 2019; Bhandage et al., 2019). Thus, from the parasite perspective, the Egr-1 responses likely contribute to maintaining the disseminatory advantage provided by shuttling DCs (Fuks et al., 2012; Kanatani et al., 2017) and efficient dissemination is assured. Additionally, we speculate that the impaired maturation of parasitized DCs may contribute to the reduction of pro-inflammatory and anti-parasitic responses (Hunter and Sibley, 2012). From the host perspective, because the Egr-1 induction is restricted to parasitized DCs, the pro-inflammatory impact is minimized. However, our data also indicate that the GRA24/p38 axis is involved in pro-inflammatory responses of DCs. It has been previously shown that macrophages secrete IL-12p40 in response to *T. gondii*-challenge in a p38- and GRA24-dependent manner (Kim et al., 2005; Braun et al., 2013). However, IL-12 produced by DCs plays a critical role in controlling acute Toxoplasma infection in mice (Mashayekhi et al., 2011). Moreover, DCs are early producers of IL-2 after bacterial challenge *in vivo* and their ability to elicit IFNγ expression in NK cells is dependent on IL-2 (Granucci et al., 2004). In this context, our data indicate that GRA24 contributes to IL-12 and IL-2 responses of DCs. Interestingly, pan-p38 inhibitor reduced IL-12 response but augmented IL-2 response. The latest is in line with observations in T cells (Kogkopoulou et al., 2006) and illustrates the cross-regulation of MAPK pathways that regulate cytokine secretion. Moreover, despite that similar Egr-1 responses were induced with both type I and II *T. gondii* lines, differences between parasite genotypes in pro-inflammatory responses await further investigation.

In the host-pathogen interplay, multiple innate immune effectors are under the control of transcription factors that modulate the complex immune responses to invasive pathogens upon infection. The data presented here adds that Egr-1 responses are tightly regulated by MAPK signaling in DCs with a down-modulatory effect on DC maturation in the context of intracellular parasitism.

## Data Availability Statement

All datasets generated for this study are included in the manuscript/Supplementary Files.

## Ethics Statement

The studies involving human participants were reviewed and approved by The Regional Ethics Committee, Stockholm, Sweden, approved protocols involving human cells. All donors received written and oral information upon donation of blood at the Karolinska University Hospital. Written consent was obtained for utilization of white blood cells for research purposes. The patients/participants provided their written informed consent to participate in this study. The animal study was reviewed and approved by The Regional Animal Research Ethical Board, Stockholm, Sweden, approved protocols involving extraction of cells from mice, following proceedings described in EU legislation (Council Directive 2010/63/EU).

## Author Contributions

AH performed experiments and analyzed data. M-AH provided crucial reagents. AH and AB conceived experimental design and wrote the manuscript.

### Conflict of Interest

The authors declare that the research was conducted in the absence of any commercial or financial relationships that could be construed as a potential conflict of interest.

## Supplementary Material

Table S1 Primer and shRNA sequences.

Figure S1 Immediate-early *Egr-1* mRNA induction leads to Egr-1 protein expression in BMDCs. Representative western blot of BMDCs challenged with freshly egressed *T. gondii* tachyzoites (PTG; MOI 5), LPS 10 ng/mL, or heat-inactivated (HI) tachyzoites for 1 h or unchallenged and probed for Egr-1 or TATA-binding protein (TBP) with densitometric analysis.

Figure S2 *Egr-*1 is induced following challenge with type I and II tachyzoites in human foreskin fibroblasts and BMDC. **(a)** Representative western blot of nucleus- (nuclear) and cytosol-enriched (cytosolic) fractions of HFFs challenged for 24 h with LPS 1 μg/mL, *T. gondii* tachyzoites (PTG MOI 1), or heat-inactivated (HI) tachyzoites (PTG MOI 1 equivalent) or unchallenged. Egr-1 signal of cytosolic fractions has a longer exposure time than nuclear fractions. **(b)** qPCR analysis of Egr-1 cDNA from BMDCs challenged for 24 h with freshly egressed PRU-RFP (type II), PTG (type II), or RH-LDM (type I) tachyzoites at MOI 1 or left unchallenged. Fold change in expression is displayed as mean ± SE (*n* = 4, ^*^*p* ≤ 0.05, ANOVA, Tukey HSD). **(c)** Representative western blot of BMDCs challenged for 24 h with freshly egressed PTG (type II), RH-LDM (type I), or PRUku80 (type II) tachyzoites (MOI 1) and probed for Egr-1 or TATA-binding protein (TBP). **(d)** qPCR analysis of Egr-2 cDNA from BMDCs challenged with freshly egressed *T. gondii* tachyzoites (PTG), LPS 10 ng/mL, heat-inactivated tachyzoites, or tachyzoite lysate for the indicated time related to unchallenged BMDCs in complete medium (CM) and area under the curve analysis thereof for the first 2 h or the whole period. Each timepoint represents the mean ± SEM of 3 independent experiments. The dashed line indicates 2 h timepoint. Bars indicate, for each condition, the cumulative *Egr-2* fold change ± SE (^*^*p* ≤ 0.05,^**^*p* ≤ 0.01, ^***^*p* ≤ 0.001, ns *p* > 0.05, permutation test).

Figure S3 IL-12p40 expression is induced in BMDCs following challenge with type I and II tachyzoites. qPCR analysis of Il12p40 cDNA from BMDCs challenged for 24 h with freshly egressed PRU-RFP (type II), PTG (type II), or RH-LDM (type I) tachyzoites at MOI 1 or left unchallenged. Relative expression (2^−ΔCq^) is displayed as mean ± SE (*n* = 4, ^*^*p* ≤ 0.05, ^**^*p* ≤ 0.01, ^***^*p* ≤ 0.001, ns *p* > 0.05, ANOVA, Tukey HSD).

Figure S4 Transduction affects BMDC differentiation and *T. gondii*-infection and LPS-challenge affect BMDC co-stimulatory markers. **(a)** Gating strategy for the experiments displayed in Figure 7. **(b)** Gating strategy for CD40, CD80, and CD86 on mock transduced BMDCs. **(c)** qPCR analysis of neutrophil elastase (Elane) cDNA from mock transduced BMDCs or BMDCs transduced with shLuc-GFP or shEgr1-GFP lentivirus. Relative expression (2^−ΔCq^) is displayed as mean ± SE (10^−3^, *n* = 6, ^*^*p* ≤ 0.05, ^**^*p* ≤ 0.01, ns *p* > 0.05, ANOVA, Tukey HSD). **(d)** Flow cytometric analysis of CD40, CD80, and CD86 expression on CD11c^+^ mock transduced and GFP^+^ shLuc- or shEgr1-transduced BMDCs that were challenged with 100 ng/mL LPS or *T. gondii* tachyzoites (PRU-RFP MOI 1) and cultured for 24 h or left unchallenged. Displayed is the mean of median fluorescence intensity of 6 independent samples (^**^*p* ≤ 0.01, ^*^*p* ≤ 0.05, ns *p* > 0.05, ANOVA, Tukey HSD).
