# Supplementary material for: Sustained Egr-1 Response via p38 MAP Kinase Signaling Modulates Early Immune Responses of Dendritic Cells Parasitized by Toxoplasma gondii
Source: Front Cell Infect Microbiol. 2019 Oct 11;9:349. doi: 10.3389/fcimb.2019.00349 (PMC6797980; doi:10.3389/fcimb.2019.00349)
Supplement: Table S1 — Primer and shRNA sequences. [file Table_1.DOCX]

| **Primers** |  |
| --- | --- |
| Target | Oligo sequence |
| Mouse Egr-1 | F: TTCAATCCTCAAGGGGAGCC  R: AAAGGACTCTGTGGTCAGGTG |
| Mouse Egr-2 | F: GAGATGGCATGATCAACATTG  R: AAGCTACTCGGATACGGGA |
| Mouse Elane | F: GTCATTTCTGTGGTGCCAC  R: CTGACCGGAAATTTAGGCC |
| Mouse Il12p40 | F: TCCCTCAAGTTCTTTGTTCG  R: CGCACCTTTCTGGTTACAC |
| Mouse Il2 | F: GGATGGAGAATTACAGGAACC  R: GAAGATCTTTCAATTCTGTGGC |
| Mouse Gapdh | F: TGACCTCAACTACATGGTCTACA  R: CTTCCCATTCTCGGCCTTG |
| Mouse Actb | F: CACTGTCGAGTCGCGTCC  R: TCATCCATGGCGAACTGGTG |
| Human EGR-1 | F: ACCGCAGAGTCTTTTCCTGA  R: GTGGTTTGGCTGGGGTAACT |
| Human IPO8 | F: GCAAAGGAAGGGGAATTGAT  R: CGAAGCTCACTAGTTTTGACCC |
| Human TBP | F: GAGCTGTGATGTGAAGTTTCC  R: TCTGGGTTTGATCATTCTGTAG |
| **shRNA oligos** |  |
| shLuc | F: TGTTCTCCGAACGTGTCACGTTTCAAGAGAACGTGACACGTTCGGAGAACTTTTTTC  R: CGAGAAAAAAGTTCTCCGAACGTGTCACGTTCTCTTGAAACGTGACACGTTCGGAGAACA |
| shEgr1 | F: CCGGCACTCCACTATCCACTATTAACTCGAGTTAATAGTGGATAGTGGAGTGTTTTTG |

**Table S1. Primer and shRNA sequences**
