# Supplementary figures and images for: Sustained Egr-1 Response via p38 MAP Kinase Signaling Modulates Early Immune Responses of Dendritic Cells Parasitized by Toxoplasma gondii
Source: Front Cell Infect Microbiol. 2019 Oct 11;9:349. doi: 10.3389/fcimb.2019.00349 (PMC6797980; doi:10.3389/fcimb.2019.00349)

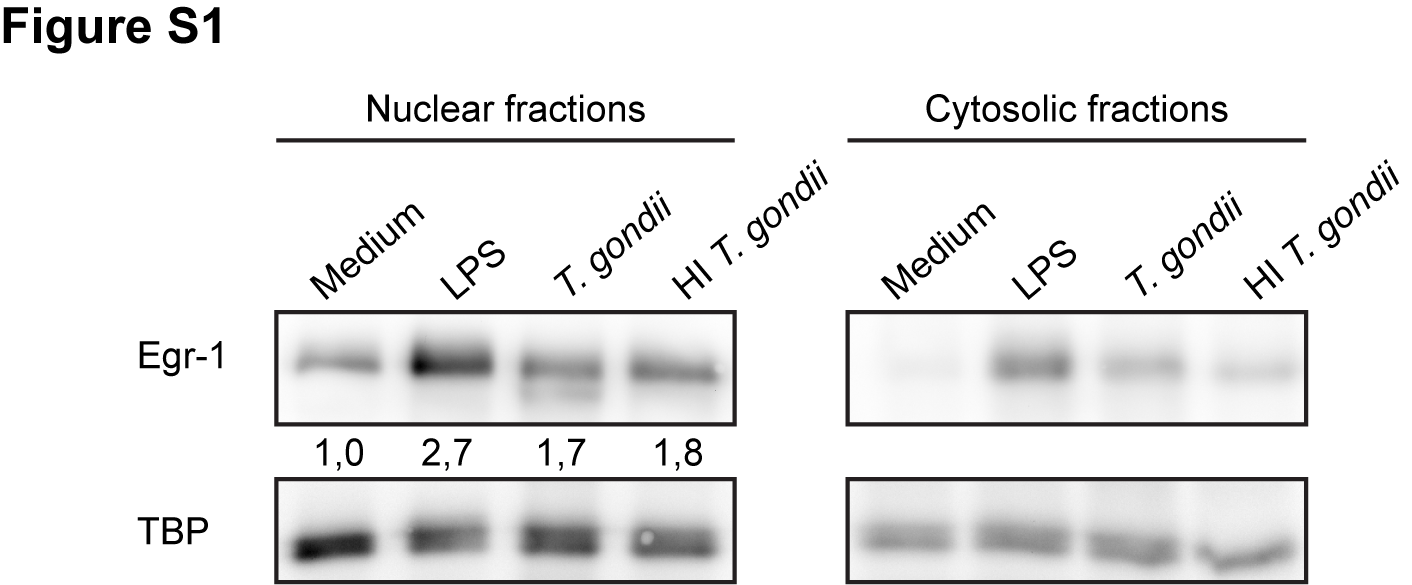

Supplement: Figure S1 — Immediate-early Egr-1 mRNA induction leads to Egr-1 protein expression in BMDCs. Representative western blot of BMDCs challenged with freshly egressed T. gondii tachyzoites (PTG; MOI 5), LPS 10 ng/mL, or heat-inactivated (HI) tachyzoites for 1 h or unchallenged and probed for Egr-1 or TATA-binding protein (TBP) with densitometric analysis. [file Image_1.TIF]

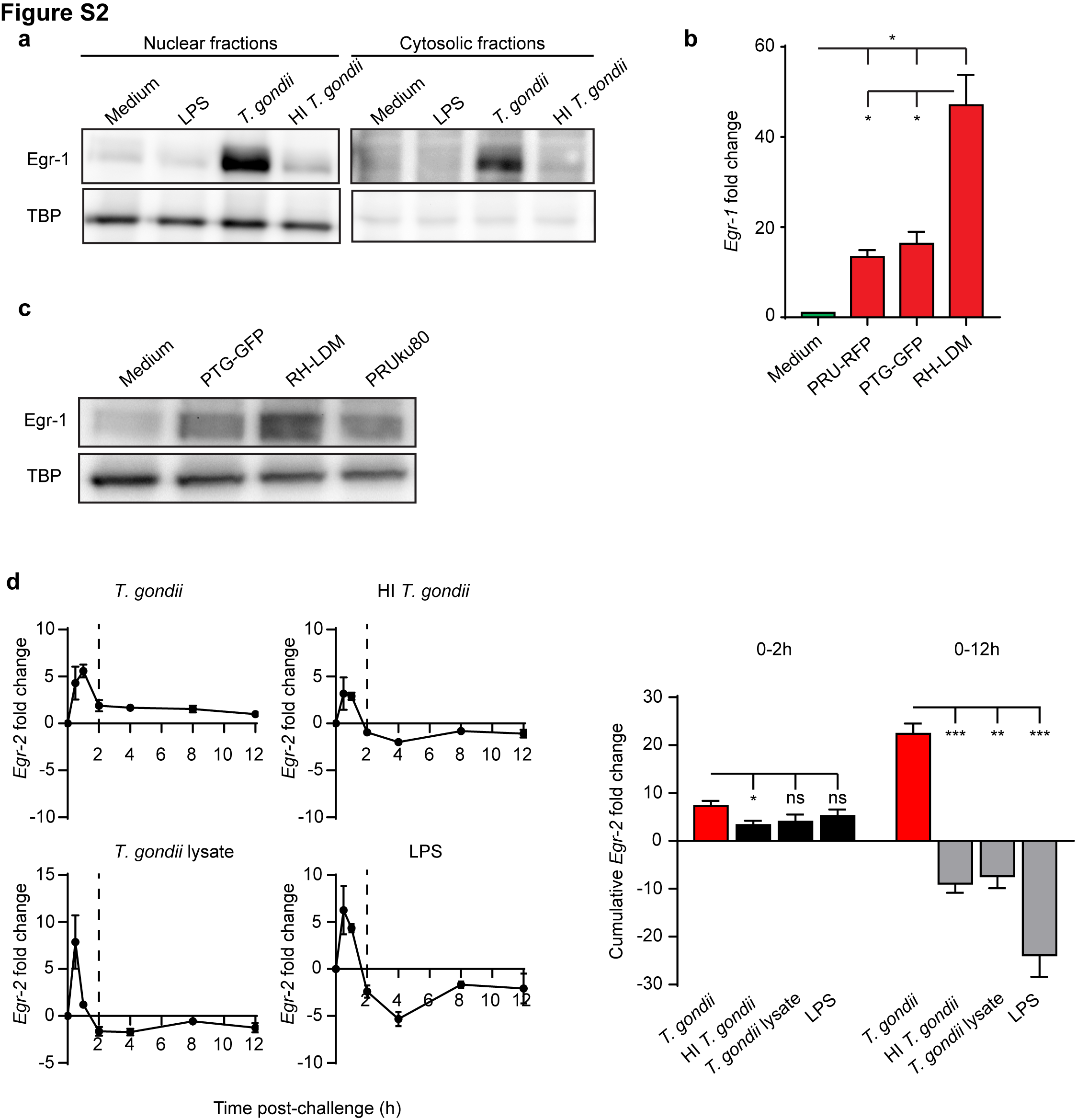

Supplement: Figure S2 — Egr-1 is induced following challenge with type I and II tachyzoites in human foreskin fibroblasts and BMDC. (a) Representative western blot of nucleus- (nuclear) and cytosol-enriched (cytosolic) fractions of HFFs challenged for 24 h with LPS 1 μg/mL, T. gondii tachyzoites (PTG MOI 1), or heat-inactivated (HI) tachyzoites (PTG MOI 1 equivalent) or unchallenged. Egr-1 signal of cytosolic fractions has a longer exposure time than nuclear fractions. (b) qPCR analysis of Egr-1 cDNA from BMDCs challenged for 24 h with freshly egressed PRU-RFP (type II), PTG (type II), or RH-LDM (type I) tachyzoites at MOI 1 or left unchallenged. Fold change in expression is displayed as mean ± SE (n = 4, *p ≤ 0.05, ANOVA, Tukey HSD). (c) Representative western blot of BMDCs challenged for 24 h with freshly egressed PTG (type II), RH-LDM (type I), or PRUku80 (type II) tachyzoites (MOI 1) and probed for Egr-1 or TATA-binding protein (TBP). (d) qPCR analysis of Egr-2 cDNA from BMDCs challenged with freshly egressed T. gondii tachyzoites (PTG), LPS 10 ng/mL, heat-inactivated tachyzoites, or tachyzoite lysate for the indicated time related to unchallenged BMDCs in complete medium (CM) and area under the curve analysis thereof for the first 2 h or the whole period. Each timepoint represents the mean ± SEM of 3 independent experiments. The dashed line indicates 2 h timepoint. Bars indicate, for each condition, the cumulative Egr-2 fold change ± SE (*p ≤ 0.05,**p ≤ 0.01, ***p ≤ 0.001, ns p > 0.05, permutation test). [file Image_2.TIF]

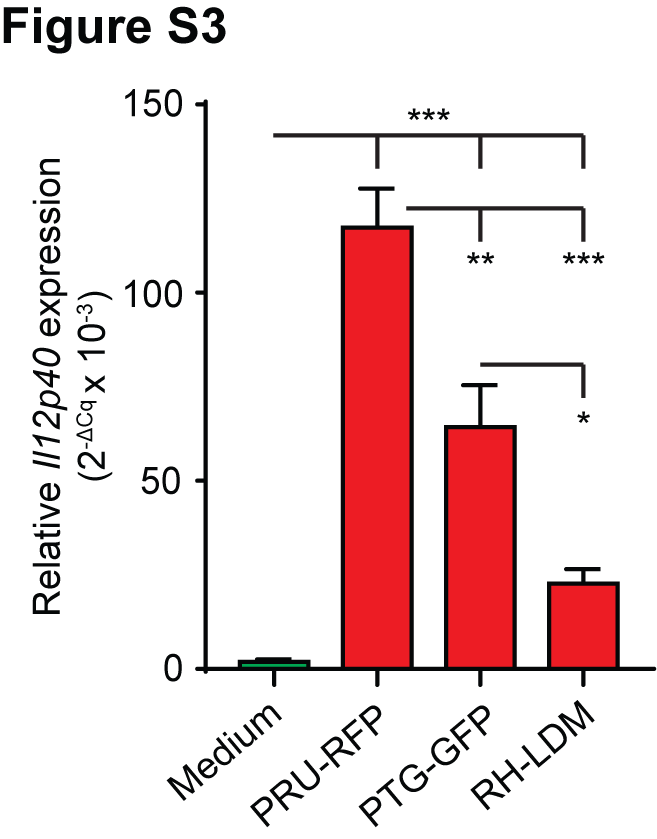

Supplement: Figure S3 — IL-12p40 expression is induced in BMDCs following challenge with type I and II tachyzoites. qPCR analysis of Il12p40 cDNA from BMDCs challenged for 24 h with freshly egressed PRU-RFP (type II), PTG (type II), or RH-LDM (type I) tachyzoites at MOI 1 or left unchallenged. Relative expression (2−ΔCq) is displayed as mean ± SE (n = 4, *p ≤ 0.05, **p ≤ 0.01, ***p ≤ 0.001, ns p > 0.05, ANOVA, Tukey HSD). [file Image_3.TIF]

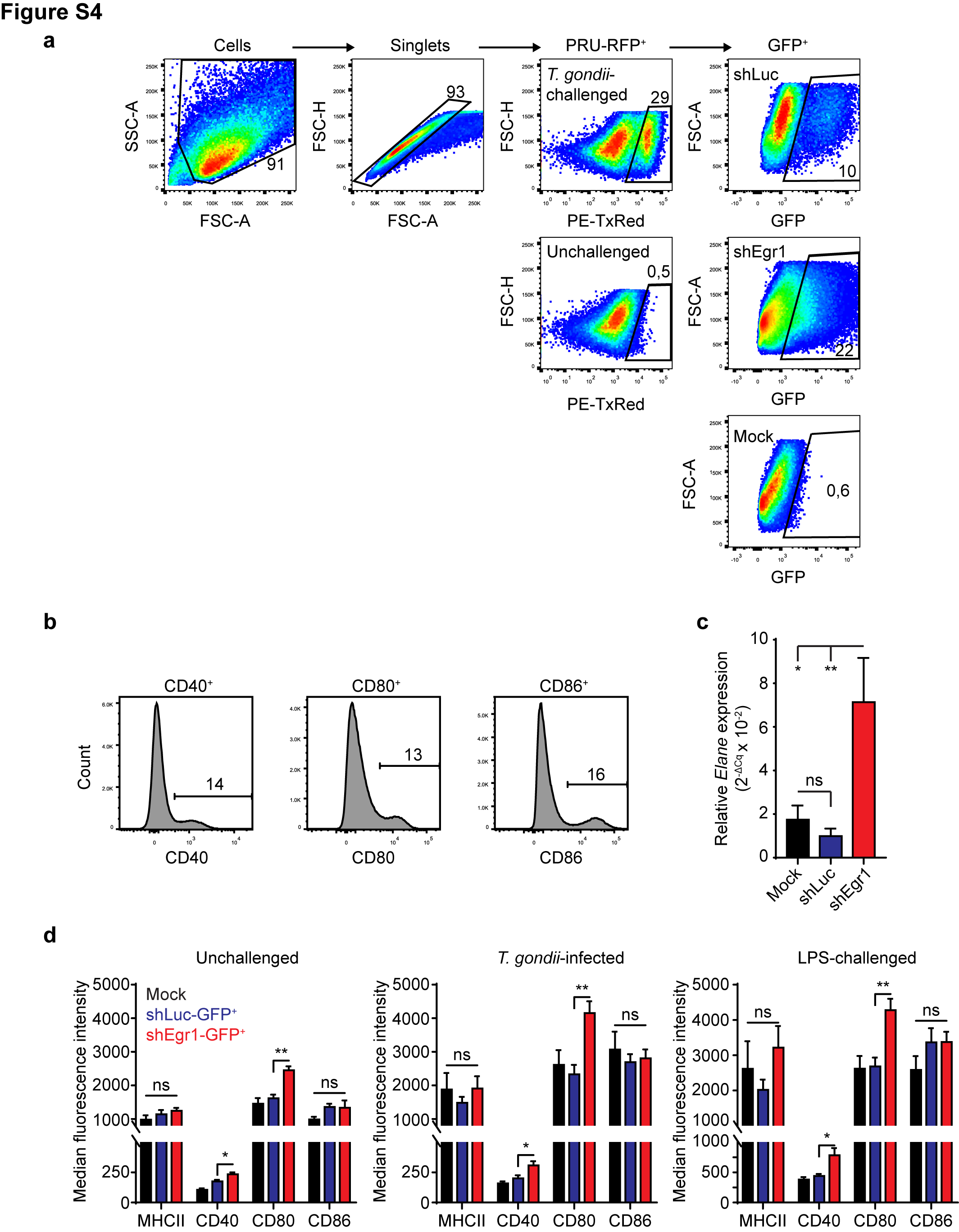

Supplement: Figure S4 — Transduction affects BMDC differentiation and T. gondii-infection and LPS-challenge affect BMDC co-stimulatory markers. (a) Gating strategy for the experiments displayed in Figure 7. (b) Gating strategy for CD40, CD80, and CD86 on mock transduced BMDCs. (c) qPCR analysis of neutrophil elastase (Elane) cDNA from mock transduced BMDCs or BMDCs transduced with shLuc-GFP or shEgr1-GFP lentivirus. Relative expression (2−ΔCq) is displayed as mean ± SE (10−3, n = 6, *p ≤ 0.05, **p ≤ 0.01, ns p > 0.05, ANOVA, Tukey HSD). (d) Flow cytometric analysis of CD40, CD80, and CD86 expression on CD11c+ mock transduced and GFP+ shLuc- or shEgr1-transduced BMDCs that were challenged with 100 ng/mL LPS or T. gondii tachyzoites (PRU-RFP MOI 1) and cultured for 24 h or left unchallenged. Displayed is the mean of median fluorescence intensity of 6 independent samples (**p ≤ 0.01, *p ≤ 0.05, ns p > 0.05, ANOVA, Tukey HSD). [file Image_4.TIF]
